# The relationship between police contacts for drug use-related crime and future arrests, incarceration, and overdoses: a retrospective observational study highlighting the need to break the vicious cycle

**DOI:** 10.1186/s12954-022-00652-2

**Published:** 2022-06-27

**Authors:** Alice Zhang, Joseph A. Balles, Jennifer E. Nyland, Thao H. Nguyen, Veronica M. White, Aleksandra E. Zgierska

**Affiliations:** 1 Department of Family and Community Medicine, Penn State College of Medicine, Hershey, PA USA; 2 City of Madison Police Department, Safe Communities Madison-Dane County, Inc., Madison, WI USA; 3 Department of Neural and Behavioral Sciences, Penn State College of Medicine, Hershey, PA USA; 4 School of Medicine and Public Health, University of Wisconsin-Madison, Madison, WI USA; 5 Department of Industrial and Systems Engineering, University of Wisconsin-Madison, Madison, WI USA

**Keywords:** Addiction, Opioid, Crime, Substance use disorder, Overdose, Incarceration

## Abstract

**Background:**

Individuals with substance use disorder often encounter law enforcement due to drug use-related criminal activity. Traditional policing approaches may not be effective for reducing recidivism and improving outcomes in this population. Here, we describe the impact of traditional policing approach to drug use-related crime on future recidivism, incarceration, and overdoses.

**Methods:**

Using a local Police Department (PD) database, we identified individuals with a police contact with probable cause to arrest for a drug use-related crime (“index contact”), including for an opioid-related overdose, between September 1, 2015, and August 31, 2016 (Group 1, *N* = 52). Data on police contacts, arrests, and incarceration 12 months before and after the index contact were extracted and compared using Fisher’s exact or Wilcoxon signed-rank tests. County-level data on fatal overdoses and estimates of time spent by PD officers in index contact-related responses were also collected. To determine whether crime-related outcomes changed over time, we identified a second group (Group 2, *N* = 263) whose index contact occurred between September 1, 2017, and August 31, 2020, and extracted data on police contacts, arrests, and incarceration during the 12 months prior to their index contact. Pre-index contact data between Groups 1 and 2 were compared with Fisher’s exact or Mann–Whitney *U* tests.

**Results:**

Comparison of data during 12 months before and 12 months after the index contact showed Group 1 increased their total number of overdose-related police contacts (6 versus 18; *p* = 0.024), incarceration rate (51.9% versus 84.6%; *p* = 0.001), and average incarceration duration per person (16.2 [SD = 38.6] to 50 days [SD = 72]; *p* < 0.001). In the six years following the index contact, 9.6% sustained a fatal opioid-related overdose. For Group 1, an average of 4.7 officers were involved, devoting an average total of 7.2 h per index contact. Comparison of pre-index contact data between Groups 1 and 2 showed similar rates of overdose-related police contacts and arrests.

**Conclusions:**

The results indicated that the traditional policing approach to drug use-related crime did not reduce arrests or incarceration and was associated with a risk of future overdose fatalities. Alternative law enforcement-led strategies, e.g., pre-arrest diversion-to-treatment programs, are urgently needed.

## Background

In 2017, the opioid epidemic in the USA was declared a national public health emergency by the Federal government [1]. At that time, approximately 1.7 million Americans had opioid use disorder (OUD) related to prescription-based opioids [2], with more than 47,000 individuals dying from a fatal overdose from either prescription-based opioids, heroin, or fentanyl [3]. Since then, the opioid epidemic has shown no sign of abating, with rates of fatal overdoses continuing to increase due largely to fentanyl being mixed into the nation's heroin supply chain [4, 5].

Connecting individuals with substance use disorder (SUD) to treatment can reduce the incidence of overdoses [6]. However, individuals with untreated SUD may come into contact with the criminal justice system before encountering the health care system [7]. Often encounters with law enforcement result from an overdose event or a criminal activity related to SUD. A significant number of individuals with OUD find themselves at some point involved with the criminal justice system. A study conducted in 2016 of US adults found that more than half of the individuals with OUD associated with prescription opioid or heroin use reported interaction with the criminal justice system within the past year [8]. A separate study conducted in Norway between 1997 and 2003 observed a higher risk of convictions among those with untreated OUD compared to those treated with medications for OUD (MOUD) [9]. In addition, treatment of prisoners with MOUD has been linked to reduced recidivism [10, 11].

The traditional policing response to drug use-related events (e.g., overdose, crime) is typically aligned with the criminal justice system approach and focuses on investigating whether there is evidence suggesting that a drug use-related crime was committed (e.g., possession of drugs or drug use-related paraphernalia). Consequently, if there is probable cause for arrest, the police, following the traditional policing approach, are tasked to arrest the individual or place charges for later arrest and criminal prosecution, which may lead to eventual incarceration. While this criminal justice system pathway can sometimes serve as an opportunity to deliver treatment to individuals during incarceration, there is an increased risk of fatal overdose following release from incarceration [12, 13]. Furthermore, incarceration per se does not appear to reduce recidivism rates or the likelihood of future incarceration for individuals with SUD [14].

Although prior studies have documented the disproportionate involvement of individuals with SUD in the criminal justice system and poor outcomes related to overdoses and re-incarceration following release from prison, not all individuals who have contact with law enforcement during a drug use-related incident are arrested or incarcerated. More research is needed on the impact of police contacts for drug use-related crime, as well as the police’s role when they interact with individuals with SUD/OUD while investigating drug use-related crime, including those related to non-fatal overdoses. There is preliminary evidence showing those with a non-fatal overdose are more likely to have been arrested for drug possession within 6 months of their overdose [15]. Another study examining outcomes following a police response to a non-fatal overdose requiring naloxone administration found that, compared to an emergency medical services (EMS) response, a police response was more likely to result in arrest immediately following the overdose response [16]. While that study did not find differences in recidivism or mortality rates over two-year follow-up among the groups who received an overdose response from the police versus EMS, it excluded responses when both police and EMS participated (which is a common scenario) and did not focus on pre–post-assessment of outcomes within each group.

Given that traditional criminal justice-based policing approaches may not be effective in reducing subsequent crime recidivism or overdoses, new policing initiatives, defined as programs differing from the traditional approach, such as pre-arrest diversion programs or assistance with treatment referrals, have been developed and are being tested to address this problem and to redirect individuals with addiction, especially OUD, from the criminal justice system to SUD treatment [17–19]. In recent years, additional overdose prevention initiatives and legislative changes (e.g., a standing order for naloxone in many states) have been implemented throughout the nation to help stem the opioid epidemic-related harms.

This study aimed to examine the relationship between a police contact for drug use-related crime within the traditional policing paradigm and future police contacts, arrests, incarceration, and overdoses. Based on the existing evidence, we hypothesized that the traditional policing approach to drug use-related crime would not be associated with reduced crime recidivism or overdoses. To test this hypothesis, we compared the rates of police contacts, arrest, and incarceration 12 months prior and 12 months after a police contact with probable cause to arrest for a drug use-related crime (“index contact”) that took place from September 2015 to August 2016 (Group 1; *N* = 52). The rate of fatal overdose following the index police contact in this group and the time spent by police officers responding to the index contact were also assessed in this group. Because the policing methods as well as the rates of police contacts, arrests, and incarceration for drug use-related crime could have spontaneously evolved over time due to changing cultural, regulatory, and political environments, we compared these outcomes of interest between Group 1 and those with an index police contact that took place from September 2017 to August 2020 (Group 2; *N* = 263) who were part of a larger project [19].

## Methods

### Design

This study’s data were collected as part of a larger, law enforcement-led project, the Madison Addiction Recovery Initiative (MARI), which took place in a medium-sized US city [19]. The city’s PD consists of approximately 480 commissioned police officers, of which approximately half are first responders or have patrol-related duties. MARI aimed at reducing crime recidivism and overdose fatalities by directing individuals who committed a drug use-related minor crime to addiction treatment in lieu of arrest and prosecution. The MARI protocol, detailed elsewhere [19], was reviewed by the Institutional Review Board and deemed not to constitute human subjects research, based on 45 CFR 46.102(d).

For this study, de-identified retrospective data on police contacts, arrests, and incarceration were provided by the local police department (PD) for eligible individuals who comprised two non-overlapping groups. Group 1 (*N* = 52) consisted of eligible adults who had a police contact with probable cause to arrest for a drug use-related crime (“index contact”) between September 1, 2015, and August 31, 2016. Group 2 (*N* = 263) was composed of adults whose index contact occurred between September 1, 2017, and August 31, 2020. For Group 1, eligible participants were identified retrospectively by a designated police officer who searched the existing local PD database for emergency call logs related to opioid overdose incidents between September 1, 2015, and August 31, 2016. At that time, the local police officers were required to report all overdose-related incidents, which were then marked in the database, enabling automatic extraction of these records; these records were then manually reviewed by the designated police officer to determine eligibility. All individuals in the overdose-related emergency call reports who met the project eligibility criteria during the assessment period were included into Group 1.

For Group 2, eligible participants were identified from September 1, 2017, to August 31, 2020, by local PD officers during their investigation. The PD officers were trained to identify and assess individuals for eligibility during their routine contacts for drug use-related crime as detailed elsewhere [19]. Individuals deemed by the police officer to be eligible and who signed the consent form to enter the pre-arrest diversion program were referred to that program [19]; their eligibility was then verified by a designated PD officer before they were enrolled into the program and formed Group 2. Police officers did not track the information on individuals who were deemed eligible but refused the program participation.

### Participants

#### Eligibility criteria

Details of eligibility criteria are described elsewhere [19]. In brief, eligible individuals were adults (at least 18 years old) who lived within the local county and had committed an eligible (“low level”) drug use-related crime. Eligible crime had to be committed within the PD’s jurisdiction, be drug use-related, and fall into one of the following categories: possession of narcotics/drugs/drug paraphernalia for personal use, prostitution, retail theft, theft from auto without property damage, burglary/theft from family members who were not pressing charges, or drug overdose with probable cause to arrest. Individuals who were on probation/parole, on bail for domestic charges or non-eligible offenses, were registered as a sex offender, had an active arrest warrant, or had a history of a violent felony conviction in the past 3 years were excluded.

Individuals in Groups 1 and 2 had the same eligibility criteria. In other words, although Group 2 consisted of individuals identified by local PD officers as candidates for the pre-arrest diversion program, individuals in Group 1 would have been eligible candidates for this program had it existed at that time. The only exclusion criteria that could not be applied to Group 1 was a parole/probation status, which was unfeasible to reliably determine due to the retrospective nature of data extraction for Group 1.

### Outcome measures

Using existing law enforcement databases, the PD-designated officer extracted and provided data on participant demographics, police contacts (number of total contacts, overdose-related contacts), arrests (number, type), and incarceration in the local county jail (number of episodes, number of days of each episode) for each individual from Group 1 (for 12 months prior and 12 months after their index contact) and Group 2 (for 12 months prior to their index contact only). Arrests were reported as the percentage of individuals who were arrested (Yes/No) and as the number of episodes across the group. Arrests were divided into three main categories based on the common classification of crimes used within the US criminal justice system: property arrest (related to “property crime,” such as theft, shoplifting, theft from auto, and damage to property), society arrest (related to “crimes against society,” such as disorderly conduct, prostitution, and drug-related offenses), and person arrest (related to “person crime,” such as homicide, sexual assault, aggravated assault, and robbery) [20]. A given individual could have been charged with several different types of crime and, as such, assigned to more than one arrest category; therefore, the sum of different arrest categories could have exceeded the total number of arrest episodes. An additional arrest category, labeled as overdose-related arrest, was created specifically for this study. It was independent of the three main arrest types. Overdose-related arrests were determined by the PD-designated officer who reviewed the local PD database for the incident type codes and reviewed the police reports to determine if the arrests were overdose-related. De-identified PD-extracted data were shared with the study team for statistical analyses.

The PD also extracted data on the number of police officers involved and the number of minutes spent by these police officers in response to each index contact for Group 1 to estimate the total time required to respond to the index contact. In addition, aggregate data on the number of opioid-related fatal overdoses that had occurred after the index contact up until March 22, 2021, were provided for Group 1 by the local public health department using the county-level mortality records [21].

### Statistical methods

Descriptive statistics summarized crime-related data for both groups, with rates of events of interest (percent affected) describing the occurrence of these events over a 12-month assessment period. Wilcoxon signed-rank and Fisher’s exact tests were used to compare pre–post-outcomes within Group 1. Mann–Whitney *U* and Fisher’s exact tests were used to compare the pre-index contact outcomes between Groups 1 and 2. Analyses were completed using the R statistical software.

## Results

A retrospective review of the PD records identified 52 eligible individuals who then formed Group 1; all index contacts for Group 1 members were related to opioid overdose. A total of 349 referrals were made to Group 2 by police officers during their investigation; after review by the designated police officer, eligibility was confirmed for 263 referred individuals who then formed Group 2, and revoked for 86 referrals. The reasons for ineligibility were: duplicate referrals (*n* = 20), currently on probation/parole (*n* = 16), self-referral without a qualifying crime (*n* = 13), ineligible criminal history or offense (*n* = 17), not being a local county resident (*n* = 7), and other various reasons (*n* = 13). Data on the number of individuals who were eligible yet declined program participation were not tracked by the police officers investigating the crime scenes. For Group 2, although many types of drug use-related crime could have been eligible for inclusion, the vast majority (93%) of the index contacts were related to opioid overdose incidents.

The majority of Group 1 (*N* = 52) members were white males, with a mean age of 30.0 (SD 9.1) years; approximately 13% of them were identified as homeless or not having a permanent address (Table 1). Group 2 (*N* = 263) members had similar demographic characteristics with the exception of age: Group 2’s mean age of 34.9 (SD 10.5) years was higher than that of Group 1 members (*p* < 0.001; Table 1).

**Table 1:** Group demographics at the time of their index contact

Demographics	Group 1 (*N* = 52)	Group 2 (*N* = 263)	*p* value
Age, years, mean (SD)	30 (9.1)	34.94 (10.5)	** < 0.001**
Women, # (%)	18 (34.6)	97 (36.9)	0.875
*Race, # (%)*			
White	46 (88.5)	210 (79.9)	0.175
Other	6 (11.5)	53 (20.2)	
Homeless, # (%)	7 (13.5)	30 (11.4)	0.641

*p* value: Mann–Whitney *U* test for continuous variables or Fisher’s exact test for categorical variables was used in comparing Group 1 and Group 2 outcomes

### Police contact, arrest, and incarceration outcomes

#### Group 1 (*N* = 52): 12 months before and after their index contact (Table 2; Fig. 1)

**Table 2:** Group 1 (*N* = 52): Police contacts, arrests, and incarceration 12 months before and 12 months after the index contact

Variable	12 months before	12 months after	*p* value
*Total police contacts*			
Yes, # (%)	30 (57.7)	36 (69.2)	0.309
# Episodes	191	167	0.488
mean (SD), median	3.7 (10.3), 1	3.2 (6.1), 1	
*Overdose-related police contacts*			
Yes, # (%)	6 (11.5)	15 (28.8)	**0.049**
# Episodes	6	18	**0.024**
Mean (SD), median	0.1 (0.3), 0	0.4 (0.6), 0	
*Total arrests*			
Yes, # (%)	24 (46.2)	26 (50.0)	0.845
# Episodes	95	84	0.841
Mean (SD), median	1.8 (5.2), 0	1.6 (3.7), 0.5	
*Overdose-related arrests*			
Yes, # (%)	4 (7.7)	3 (5.8)	1.000
# Episodes	4	5	1.000
Mean (SD), median	0.1 (0.3), 0	0.1 (0.5), 0	
*Person arrests*			
Yes, # (%)	4 (7.7)	4 (7.7)	1.000
# Episodes	12	5	0.750
Mean (SD), median	0.2 (1.1), 0	0.1 (0.4), 0	
*Society arrests*			
Yes, # (%)	19 (36.5)	24 (46.2)	0.426
# Episodes	64	65	0.604
Mean (SD), median	1.2 (4.1), 0	1.3 (3.3), 0	
*Property arrests*			
Yes, # (%)	10 (19.2)	5 (9.6)	0.264
# Episodes	19	14	0.423
Mean (SD), median	0.4 (0.8), 0	0.3 (1.0), 0	
*Incarceration*			
Yes, # (%)	27 (51.9)	44 (84.6)	**0.001**
# Days	842 days	2600 days	** < 0.001**
Mean (SD), median	16.2 (38.6), 1	50.0 (72.0), 13	

*p* value: Wilcoxon signed-rank test for continuous variables or Fisher’s exact test for categorical variables were used in comparing pre- versus post-index contact outcomes

**Fig. 1:**
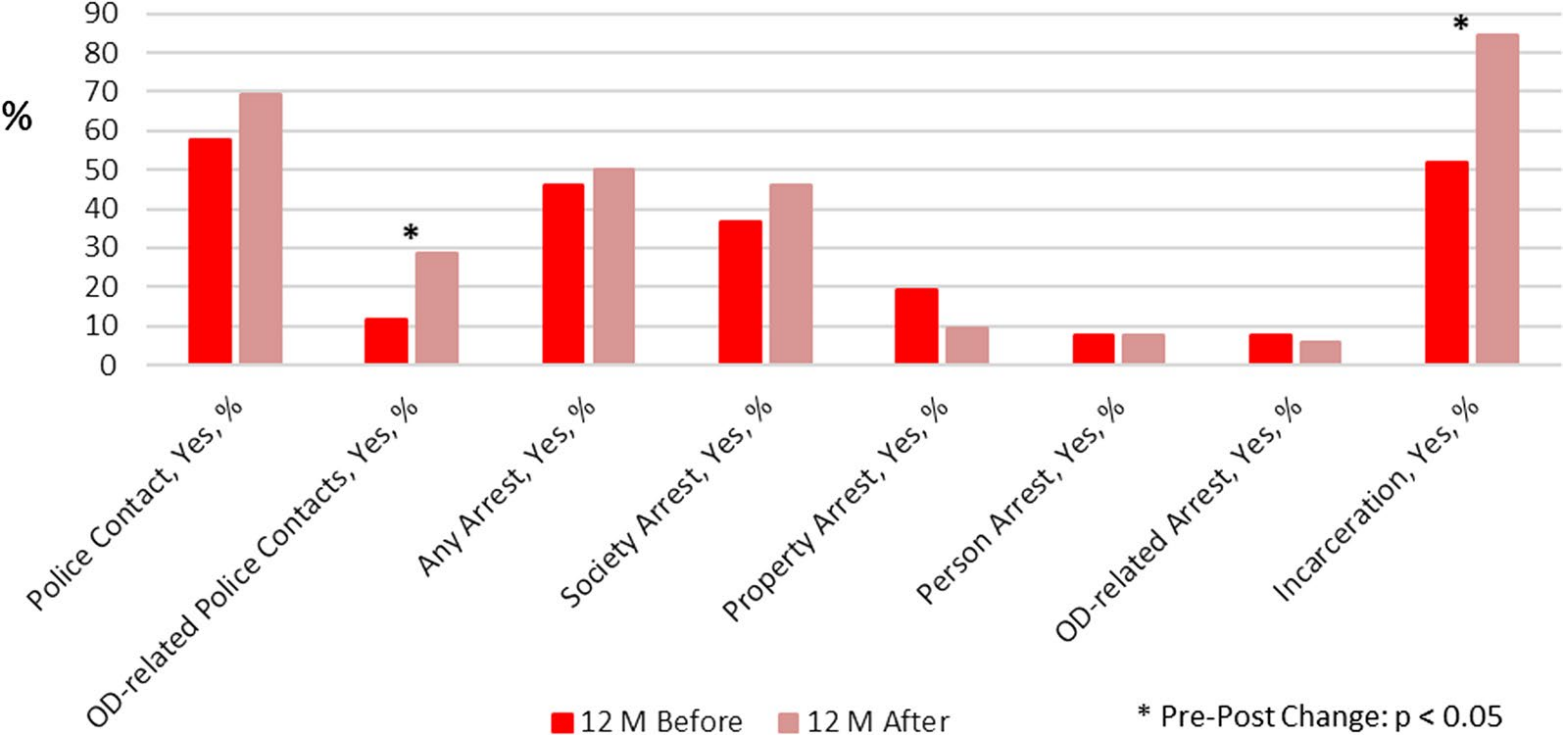
Group 1 (*N* = 52): Police contacts, arrest, and incarceration 12 months before and after the index contact. OD: overdose

#### Group 2 (*N* = 263): 12 months before their index contact (Table 3)

In the 12 months prior to their index contact, the majority (57.7%) of Group 1 had at least one police contact, totaling 191 contacts across the group, with 6 of these contacts for opioid-related overdose. In addition, 46.2% of them had been arrested, with a total of 95 arrests in the group. In Group 1, society arrests (36.5%) were most common, followed by property (19.2%) and person (7.7%) arrests; 7.7% of all arrests were identified as overdose-related. Slightly over half (51.9%) of Group 1 members had been incarcerated, with a total of 842 days spent in jail across the group and averaging 16.2 (SD 38.6) days of jail time per person (median = 1).

**Table 3:** Police contacts, arrests, and incarceration outcomes during 12 months before the index contact in Group 1 (*N* = 52) and Group 2 (*N* = 263)

Variable	Group 1 (*N* = 52)	Group 2 (*N* = 263)	*p* value (Group 1 vs 2)
*Total Police contacts*			
Yes, # (%)	30 (57.7)	167 (63.5)	0.437
# Episodes	191	633	
Mean (SD)	3.7 (10.3)	2.4 (3.4)	0.346
Median	1	1	
*OD-related contacts*			
Yes, # (%)	6 (11.5)	38 (14.4)	0.667
# Episodes	6	46	
Mean (SD)	0.1 (0.3)	0.2 (0.5)	0.554
Median	0	0	
*Total arrests*			
Yes, # (%)	24 (46.2)	82 (31.2)	0.053
# Episodes	95	171	
Mean (SD)	1.8 (5.2)	0.7 (1.4)	**0.024**
Median	0	0	
*OD-related arrests*			
Yes, # (%)	4 (7.7)	17 (6.5)	0.761
# Episodes	4	22	
Mean (SD)	0.1 (0.3)	0.1 (0.4)	0.759
Median	0	0	
*Person arrests*			
Yes, # (%)	4 (7.7)	12 (4.6)	0.313
# Episodes	12	13	
Mean (SD)	0.2 (1.1)	0.1 (0.2)	0.327
Median	0	0	
*Society arrests*			
Yes, # (%)	19 (36.5)	56 (21.3)	**0.031**
# Episodes	64	109	
Mean (SD)	1.2 (4.1)	0.4 (1.0)	**0.019**
Median	0	0	
Property arrests			
Yes, # (%)	10 (19.2)	26 (9.9)	0.060
# Episodes	19	49	
Mean (SD)	0.4 (0.8)	0.2 (0.7)	**0.048**
Median	0	0	
*Incarceration*			
Yes, # (%)	27 (51.9)	76 (28.9)	**0.002**
Days, #	842	2683	
Mean (SD)	16.2 (38.6)	10.2 (31.6)	**0.001**
Median	1	0	

*p* value: Mann–Whitney *U* test for continuous variables or Fisher’s exact test for categorical variables was used in comparing Group 1 and Group 2 outcomes

Compared to the prior 12 months, during the 12 months following their index contact, Group 1 increased the rate of overdose-related police contacts (11.5% versus 28.8%; *p* = 0.049), the total number of overdose episodes (6 versus 18; *p* = 0.024), the rate of incarceration (51.9% versus 84.6%; *p* = 0.001), and the mean incarceration duration per person (16.2 [SD 38.6] versus 50.0 [SD 72.0]; *p* < 0.001). The number of total police contacts and arrests did not change in a statistically significant way from pre- to post-index contact period.

In the 12 months prior to their index contact, the majority (63.5%) of Group 2 members had at least one police contact, with 14.4% related to overdose. Approximately one-third (31.2%) of Group 2 had been arrested, with the majority being arrested for society arrests (21.3%), followed by property (9.9%) and person (4.6%) arrests. In Group 2, 6.5% of its members had an overdose-related arrest. Fewer than one-third of Group 2 members (28.9%) had been incarcerated, spending a total of 2,683 days in jail across the group and averaging 10.2 (SD 31.6) days per person (median 0).

#### Comparison of Group 1 and Group 2 outcomes: 12 months before the index contact (Table 3)

Comparison of Group 1 and 2 outcomes in the 12 months prior to their index contact showed the two groups did not differ (*p* ≥ 0.05) in the total and overdose-related police contacts. Compared to Group 2, Group 1 trended toward a higher rate of arrests (*p* = 0.053) and a higher mean number of arrest episodes per person (1.8 versus 0.7; *p* = 0.024), with society and property arrest types driving these between-group differences (*p* < 0.05). Group 1 also had higher incarceration rates (51.9% vs. 28.9%; *p* = 0.002) and a longer average incarceration period (16.2 ± 38.6 versus 10.2 ± 31.6 days; *p* = 0.001).

### Fatal overdoses (Group 1)

As of March 22, 2021, five individuals in Group 1 (9.6%) had died due to a fatal overdose: two within the initial 12 months after the index contact, and the remaining three occurred more than 12 months after the index contact.

### Time spent by law enforcement in response to index contacts (Group 1)

Data on police officer involvement when responding to overdose-related index contacts were missing for one Group 1 member; therefore, the following estimates were based on the data available for index contacts for 51 individuals. For these 51 individuals, 238 police officers were involved in the overdose-related response, averaging 4.7 officers per index contact. These officers logged a total of 366.4 h, averaging 7.2 h per overdose-related index contact.

## Discussion

### Interpretation

Our findings indicate that a traditional policing approach relying on a criminal justice pathway to drug use-related crime, particularly those related to non-fatal overdose, did not lead to reductions in crime recidivism, arrest, or incarceration. When looking at incarceration outcomes, individuals with overdose-related police contacts between September 2015 and August 2016 (Group 1) experienced notable increases in both the incarceration rate and average incarceration duration per person in the 12 months following their index contact. The average incarceration duration per person in Group 1 nearly tripled, and this increase did not seem to be driven by a few outliers only; the vast majority of Group 1 (84.6%) experienced incarceration in the year following their overdose-related index contact, with ten (19.2%) incarcerated for at least 100 days. The longitudinal outcomes observed within Group 1 were consistent with prior studies demonstrating that incarceration does not have a positive impact in reducing recidivism or overdose rates [12–14, 22].

In addition, the percentage of individuals with police contacts for non-fatal overdoses and the number of such contacts increased for Group 1 in the 12 months following the index contact. This increase occurred despite two individuals dying of a fatal overdose within the 12-month post-index contact time frame. It is possible the rate of police contacts for non-fatal overdoses and the rates of arrests and incarceration in the 12 months after the index contact may have been even higher than currently reported if the two individuals had not sustained fatal overdoses within that period. A different policing approach at the time of the index (or subsequent) contacts for non-fatal overdose could have prevented the fatal occurrences. Overall, our results document an individual’s increasing struggles with addiction as we observed increasing trends with arrest, incarceration, and overdose events following an overdose-related police contact under the criminal justice-based policing method.

Collecting data or measures on arrest and incarceration is a fairly simple task on the surface, but at times can be more difficult to analyze, interpret, and understand. Although an arrest typically precedes incarceration, in this study we did not observe statistically significant increases in the total arrests in Group 1 from pre- to post-index contact, despite the dramatically worsened incarceration-related outcomes. This could be explained by the different sources of our data; the arrest data were available from one local PD’s database, while the incarceration data stemmed from a county jail, which receives inmates from multiple PDs. As a result, an individual in Group 1 may have been arrested by a different PD (data unavailable for our analyses) and then incarcerated at the county jail (data extracted by our team), thus accounting for the discrepancy in arrest and incarceration rates post-index contact. Hence, Group 1’s arrest rates may have been higher than noted in this analysis.

Furthermore, the changing environment during Group 1’s “enrollment” period, including evolving law enforcement’s attitudes toward addiction and its role in preventing opioid-related overdose deaths, coupled with the nationwide sharp increase in opioid-related overdoses, may have impacted the incarceration duration during the post-index contact one-year follow-up period. From 2013 to 2015, the number of law enforcement contacts associated with fentanyl (or its analogues) misuse spiked, resulting in an increased number of overdoses [5]. Additionally, some law enforcement occasionally viewed arrest as the best option to help those with SUD [23]. Thus, in light of a rapid increase in fentanyl-related fatal overdoses, the culture within law enforcement at the time may have favored a prolonged incarceration for individuals with OUD. In the subsequent years, knowledge about increased risk of fatal overdoses following incarceration [12–14] and perspectives on the role of incarceration for individuals with SUD within the criminal justice system have continued to evolve, possibly contributing to a reduction in the incarceration duration in the following years, as seen during Group 2’s pre-index contact assessment period.

When comparing the initial sample (Group 1) to the “replication sample” (Group 2) whose index contacts occurred later (from September 2017 through August 2020), the two groups had similar rates of overdose-related police contacts and arrests prior to their index contact, despite a time gap of at least 2 years between the groups’ index contact events, and substantial local and national efforts to reduce opioid-related overdoses and OUD/SUD-related harm during that time. These efforts included an overdose prevention campaign by the State of Wisconsin’s Attorney General launched in 2015 [24] and the implementation of a standing order for naloxone in August 2016. Overall, while SUD treatment and naloxone availability have increased, there are still substantial barriers in ensuring that individuals with OUD have access to naloxone [25, 26] and evidence-based addiction treatment [27]. This is particularly important for people with OUD who are incarcerated. Jails and prisons are environments of enforced abstinence, and individuals with OUD, unless appropriately treated, often face challenges in maintaining abstinence upon release, with increased risk of relapse and overdose [14]. The risk of fatal overdose is substantially increased following a period of abstinence (such as incarceration) due to decreased tolerance [12, 13]. In Group 1, approximately 10% died from a fatal overdose within 6 years of their index contact, further corroborating the need for novel approaches to supplement the existing ones in an effort to stem the opioid epidemic and reduce overdose fatalities.

Interestingly, Group 1 had a higher incarceration rate and duration, and number of arrests during the 12 months before their index contact, compared to Group 2. These differences could be explained by different eligibility criteria regarding the parole/probation status applied to these groups. Group 2 consisted of individuals enrolled in the pre-arrest diversion program, which excluded individuals who were on probation or parole. This exclusionary criterion was not applied to individuals in Group 1 due to our inability to retrospectively and reliably determine their parole or probation status at the time of their index contact using the law enforcement database. Therefore, Group 1 members could have been on parole or probation at the time of their “study enrollment,” potentially impacting the degree of their prior (pre-index contact) criminal activity. While this may limit our ability to directly compare arrest or incarceration rates *between* the two groups, it did not impact the results stemming from comparison of *within* Group 1 changes (i.e., pre- versus post-index contact).

We additionally noted a substantial degree of police engagement when responding to the overdose-related incidents. In our analysis, an average of 4.7 officers devoted an average total of 7.2 h in response to one index contact. Despite being time consuming, the outcomes observed in our study suggest the time spent with an index contact under the traditional policing paradigm may not be effective. In addition, the above estimates did not take into account the time spent responding to an index contact by other first responders (e.g., fire department or EMS) who typically get involved in an overdose response when it is dispatched as an emergency “pulseless non-breather” call. They also do not factor in efforts by other agencies involved in the response of overdose events (e.g., medical evaluation in the Emergency Department, potential hospitalization, coroner’s office effort for fatal overdoses, the criminal justice system impact), or other PD efforts (e.g., related to the transportation or vehicle maintenance). Examining the amount of time spent responding to drug use-related crime and the associated cost is essential for cost–benefit analyses, which are useful when considering new methods or interventions. It is especially important when considering novel policing approaches, such as pre-arrest diversion-to-treatment programs, for people who committed drug use-related crime; the majority of incarcerated individuals or those on probation/parole would benefit from SUD treatment and the overall cost associated with their incarceration and probation/parole was estimated to cost the State of Wisconsin $719 million from 2015 to 2016 [28].

Thus, given the lack of documented benefit of arrest and incarceration toward reducing overdose or crime recidivism and the substantial amount of time spent by law enforcement in responding to drug use-related crime, alternative interventions should be considered for individuals with OUD who are arrested for a crime fueled by their addiction. Traditional criminal justice pathways of arrest, prosecution, and incarceration with enforced abstinence have not been effective in slowing the opioid epidemic. Rather, treatment for addiction, including for OUD, is supported by a strong evidence base, indicating dramatic reductions in overdose fatalities, decrease in crime and its recidivism, among other benefits [29–31]. Potential alternative interventions should consider diversion-to-treatment programs, provision of MOUD in prisons or jails (as indicated based on medical assessment), or reentry programs to facilitate the transition back into the community [14]. Such interventions should be comprehensive, focus on the biology of addiction, and leverage extensive evidence base on the benefits of addiction treatment. Partnerships between treatment programs, recovery community organizations, and the criminal justice system should be encouraged, given the likelihood of criminal history among individuals with OUD. In addition, arrests and incarceration related to drug use can have a substantial negative impact on employment or family stability and contribute to the propagation of disenfranchisement of already-marginalized populations [32].

## Limitations

The generalizability of our results could be limited by several factors. Individuals in both groups had contact with the police as a result of their substance use-related crime with probable cause for arrest; furthermore, the index contact in both groups was largely related to overdose-related incidents, and individuals in Group 2 were selected by the police officers for a pre-arrest diversion-to-treatment program. As an outcome, our findings may not be generalizable to the broader population of individuals with OUD who have not come into contact with law enforcement, who have had contacts with law enforcement for other “eligible” drug use-driven crime (e.g., theft, prostitution), who were ineligible based on the type of crime committed or their criminal status (e.g., being on parole/probation), or who were uninterested in addiction treatment. Furthermore, opioid-related overdoses could have occurred due to a variety of reasons, such as more severe OUD, a decreased tolerance to opioids (e.g., post-release), use of illicit opioids or other drugs with fentanyl or its analogs, or use of other sedating substances. While we were unable to delineate the causes of overdose among individuals comprising Groups 1 and 2, evidence suggests that individuals with greater opioid use are more likely to be involved with the criminal justice system [8].

In addition, Group 1 likely had a lower access to addiction treatment, especially MOUD, while incarcerated, because the vast majority of prisons and jails did not offer access to MOUD at that time. Although improvements have been made more recently, there are still significant gaps in addiction care, including MOUD, during and after incarceration [33, 34]. Regardless of the surrounding circumstances, an opioid-related overdose, which requires an emergency response by the police, represents a significant event within an individual’s life and could be seen as an opportunity for intervention and referral to treatment, thereby altering the untreated addiction trajectory.

Other limitations include a convenience sampling and the retrospective observational design as well as a subpar representation of minorities and females in our samples. This pragmatic study did not apply sophisticated sampling or selection strategies; rather, its recruitment and enrollment processes reflected a pragmatic approach to program implementation in the real-world settings. Our samples also had a subpar representation of minorities and females in our samples. Future studies with greater inclusion of underrepresented minority groups are needed, especially in light of racial inequalities associated with the criminal justice system [35], and because minorities have been disproportionately affected by the opioid crisis. From 2011 to 2016, African-Americans had the highest increase in overdose death rates due to synthetic opioids [36]. Finally, although we anticipated a similar scope of police officer involvement in their response to index contacts in both groups, it was unfeasible to extract data on the details of police officer involvement for Group 2 participants.

## Conclusions

This analysis showed that the traditional policing approach, which typically involves prosecution and arrest for drug use-related minor crime, did not reduce future arrests and incarceration. It was also associated with a risk of future fatal overdose. Furthermore, police response to overdose-related incidents required a substantial amount of police officers’ time. Alternative strategies, such as pre-arrest diversion-to-treatment programs and community policing, should be considered to direct individuals who committed drug use-related crime to addiction treatment in order to help curb the opioid epidemic and its deleterious impact.

## Data Availability

The datasets used and/or analyzed during the current study are available from the corresponding author upon reasonable request.

## Abbreviations

EMS: Emergency Medical Services
MARI: Madison Addiction Recovery Initiative
MOUD: Medications for opioid use disorder
PD: Police Department
OUD: Opioid use disorder
SUD: Substance use disorder
US: United States
