# Correction: The relationship between police contacts for drug use‑related crime and future arrests, incarceration, and overdoses: a retrospective observational study highlighting the need to break the vicious cycle

**DOI:** 10.1186/s12954-022-00669-7

**Published:** 2022-07-29

**Authors:** Alice Zhang, Joseph A. Balles, Jennifer E. Nyland, Thao H. Nguyen, Veronica M. White, Aleksandra E. Zgierska

**Affiliations:** 1 Department of Family and Community Medicine, Penn State College of Medicine, Hershey, PA USA; 2 City of Madison Police Department, Safe Communities Madison-Dane County, Inc., Madison, WI USA; 3 Department of Neural and Behavioral Sciences, Penn State College of Medicine, Hershey, PA USA; 4 School of Medicine and Public Health, University of Wisconsin-Madison, Madison, WI USA; 5 Department of Industrial and Systems Engineering, University of Wisconsin-Madison, Madison, WI USA

## Correction to: Harm Reduction Journal (2022) 19:67 https://doi.org/10.1186/s12954-022-00652-2

Following the publication of the original article [1], the text *'Group 2 (N* = *263): 12 months before their index contact (Table 3)'* was misplaced wrongly. The correct insertion of the text is as follows:

***Group 2 (N = 263): 12 months before their index contact (Table 3)***

In the 12 months prior to their index contact, the majority (63.5%) of Group 2 members had at least one police contact, with 14.4% related to overdose. Approximately one-third (31.2%) of Group 2 had been arrested, with the majority being arrested for society arrests (21.3%), followed by property (9.9%) and person (4.6%) arrests. In Group 2, 6.5% of its members had an overdose-related arrest.

The original article has been corrected.

